# Incretin-Based Therapies: A Novel Pathway in Addiction Treatment

**DOI:** 10.3390/jcm15041613

**Published:** 2026-02-19

**Authors:** Rosiejka Dawid, Michałowska Joanna, Marcickiewicz Justyna, Adela Bogdańska, Wiktoria Błażejewska, Monika Szulińska

**Affiliations:** 1 Department of Obesity Treatment, Metabolic Disorders and Clinical Dietetics, Poznan University of Medical Sciences, 61-701 Poznan, Poland; 2 Medical Education, Wroclaw Medical University, 50-367 Wroclaw, Poland; 3 Doctoral School, Poznan University of Medical Sciences, 60-812 Poznan, Poland

**Keywords:** incretin-based therapy, GLP-1, addiction treatment, alcohol dependency, nicotine addiction, substance abuse

## Abstract

Addiction poses a major global public health challenge. It is characterized by high prevalence, chronic relapse and limited efficacy of available pharmacotherapies across different substance use disorders. Increasing evidence demonstrates that incretin-based therapies directly modulate metabolic signaling pathways that intercross with central reward and motivational circuits, including hypothalamic-mesolimbic networks and dopaminergic neurotransmission. As a result, agents such as glucagon-like peptide 1 receptor agonists, originally developed for the treatment of type 2 diabetes and obesity, are now being actively investigated for their role in addiction treatment. This narrative review summarizes the current knowledge on the role of incretin-based therapies in the neurobiology of addiction. Evidence from preclinical models and human studies supports the potential therapeutic effect of glucagon-like peptide 1 receptor agonists in the treatment of alcohol use disorder, nicotine dependence, and the administration of other psychoactive substances, including psychostimulants, opioids, and cannabinoids. Preclinical studies consistently demonstrate that glucagon-like peptide 1 receptor agonists reduce substance intake, attenuate reward-related behaviors, and suppress relapse-like responding. So far, human evidence remains limited and is largely derived from observational studies. Preliminary research suggests potential reductions in substance use severity and overdose risk among individuals treated with incretin-based agents. While these findings highlight incretin signaling as a promising therapeutic option in addiction, the current evidence is insufficient to support their routine clinical use in the treatment of substance dependence. Therefore, further research is required to clarify underlying mechanisms and establish clinical efficacy. In particular, well-designed randomized controlled trials are needed to determine safety, tolerance and effectiveness of incretin-based therapies across different substance use disorders.

## 1. Epidemiology of Addiction

Addiction refers to a chronic condition characterized by compulsive substance use or behavior despite harmful consequences. It is often accompanied by tolerance, dependence, and withdrawal when the substance or behavior is reduced or stopped. Most common addictions concern alcohol, tobacco and illicit drugs. High prevalence, serious health consequences, mortality, and heavy social and economic costs make it an urgent public health problem. Globally, about 20% of adults–over one billion people, smoke tobacco [1]. At the same time, around 400 million individuals suffer from alcohol use disorder [2]. Furthermore, an estimated 64 million people worldwide (approx. 1 in 81 adults) suffer from a drug use disorder [3]. Europe stands out globally for tobacco use, alcohol consumption per capita as well as alcohol-related harm, while North America and Oceania account for a large number of people using drugs [1,2,4].

Gender influences vulnerability to substance use, with men generally exhibiting higher rates of use and substance-related health issues [2]. Age is also critical, as adolescents who initiate drinking or drug use early are significantly more likely to develop addiction in adulthood. Socioeconomic disadvantage, including low income and lower education, substantially increases the likelihood of tobacco, alcohol, and drug use due to chronic stress and reduced access to resources [5]. Furthermore, mental health conditions, particularly depression and anxiety, greatly heighten the risk of substance use [2,6].

The health impact of addiction covers a wide spectrum, including chronic diseases (e.g., cardiovascular, respiratory, liver disease, cancers), mental health disorders, and infectious diseases [1,2,7,8]. Alcohol and tobacco are classified as cancerogenic substances by the International Agency for Research on Cancer [9]. Addictions are responsible for millions of deaths annually. Tobacco alone leads to around 8 million deaths per year worldwide. Alcohol is responsible for about 2.6 million deaths, and psychoactive drug use contributes to roughly 0.6 million deaths per year [2]. According to global analysis in 2019, tobacco smoking accounts for about 229.7 million disability-adjusted life years (DALYs) [10], alcohol for roughly 115.9 million, and illicit drugs for about 36.7 million [2].

The economic burdens of addictions are enormous. Lost productivity, healthcare costs, and reduced quality of life affect not only individuals but entire societies. In the European Union (EU), healthcare costs for alcohol-related cancers were estimated at nearly 5 billion euros in 2018 [11], whereas alcohol-related harm costs England £27.44 billion every year [12]. According to the latest data from the World Health Organization (WHO) the number of tobacco users is declining worldwide [1]. Alcohol consumption in Europe has decreased since 2000, but in the Asia region it tends to increase [2]. For illicit drugs, the most recent estimates suggest a modest rise in the global proportion of adults with drug use disorders [4]. Contemporary modes of delivery and types of addictive substances are rapidly evolving, generating new and increasingly complex challenges. In modern societies, the brain’s reward system is exposed to frequent and powerful sources of dopamine activation, such as ultra-processed foods, targeted advertising, online gambling, highly accessible e-cigarettes and stronger synthetic opioids, all of which provide intensified forms of reward [13]. These emerging challenges call for innovative and more effective systemic responses to prevent the broad spectrum of harms associated addictive substances. Increasing evidence demonstrates that incretin-based therapies directly modulate metabolic signaling pathways that intercross with central reward and motivational circuits [14]. The aim of this narrative review is to summarize the current knowledge on the role of incretin hormones in addiction treatment.

## 2. Literature Search Methodology

To identify publications relevant to the aims of this narrative review, a targeted literature search was performed across four widely used scientific databases: PubMed, Scopus, ScienceDirect, and Google Scholar. The search was limited to peer-reviewed articles published in English and emphasized studies that advanced current knowledge on the role of incretin-based therapies in the context of addiction and substance use disorders.

The search strategy employed a broad set of keywords combined with Boolean operators to ensure adequate coverage of the topic. Search terms included, among others: “incretin-based therapies,” “GLP-1 receptor agonists,” “DPP-4 inhibitors,” “GLP-1,” “addiction,” “substance use disorder,” “drug dependence,” “alcohol use disorder,” and “reward pathways,” together with closely related terms and alternative expressions. Publications published to December 2025 were eligible for consideration. Studies were selected based on their relevance to the thematic scope of the review, with preference given to articles offering experimental evidence, mechanistic insights, or clinically meaningful perspectives. Although this review does not follow a formal systematic methodology, the applied search strategy was designed to ensure a broad and representative synthesis of the existing literature.

## 3. Incretins

Glucagon-like peptide-1 (GLP-1) analogs represent one of the most innovative classes of metabolic therapies developed in recent decades. These drugs are synthetic forms of the endogenous gut hormone responsible for enhancing glucose-dependent insulin secretion, reducing glucagon release, slowing gastric emptying, and promoting satiety. Initially designed for the management of type 2 diabetes (T2D), GLP-1 analogs have rapidly expanded to include long-term treatment of overweight and obesity [15]. These drugs have gained remarkable popularity globally. In the United States, approximately 13% of adults have reported using a GLP-1 receptor agonist (GLP-1RA) at some point [16]. Moreover, GLP-1 receptors (GLP-1Rs) are expressed in numerous tissues beyond the pancreas, suggesting broader therapeutic potential [17].

### 3.1. History

The first evidence of insulinotropic gut hormones was reported by La Barre in 1930, when he demonstrated that duodenal extracts lowered blood glucose in vitro, without exocrine pancreas effect, proposing that this glucose reduction might be mediated through triggering a mechanism of insulin secretion [18]. In 1932, La Barre introduced the term “incretin” to describe a gut-derived factor capable of stimulating endocrine pancreatic secretion, and he suggested that such hormones might one day provide a therapeutic approach for diabetes [19,20]. Approximately three decades later, the advent of radioimmunoassay technologies by Berson and Yalow [21] enabled scientists to examine the differential insulin response to oral versus intravenous glucose, thereby establishing what became known as the incretin effect [22,23]. This led to the identification of the first incretin hormone–gastric inhibitory polypeptide (GIP) by John Brown [24]. In the 1970s, as its physiological role became better understood, its name was changed to glucose-dependent insulinotropic polypeptide to highlight its predominant endocrine action [25]. During the 1980s, Graeme Bell and colleagues elucidated the structure of proglucagon, discovering several previously unrecognized peptides, including GLP-1 and GLP-2, which are secreted from the intestinal mucosa [26,27]. GLP-1, in particular, exhibited potent pancreatic effects, stimulating insulin secretion while suppressing glucagon release. Nearly a decade later, Drs. John Eng and Jean-Pierre Raufman isolated exendin-4 (EX-4) from the venom of the Gila monster, a peptide with GLP-1–like activity but a substantially longer half-life [28,29]. Dr. John Eng subsequently licensed the compound and developed a synthetic analogue, ultimately resulting in exenatide, which received Food and Drug Administration (FDA) approval in 2005 for the treatment of T2D [30]. In 2009 liraglutide was introduced, a human GLP-1 analogue that enabled once-daily administration and became the first incretin therapy approved for both T2D and obesity. This was followed by the approval of once-weekly dulaglutide in 2014, and its equivalent semaglutide in 2017. In 2019, oral semaglutide was launched, marking the first orally administered GLP-1RA [31]. Subsequently, in 2022, a dual GLP-1/GIP receptor agonist was developed, offering enhanced metabolic efficacy compared with earlier single-agonist agents [32]. Currently, intensive research is focused on triple agonists targeting GLP-1, GIP, and glucagon receptors demonstrating unprecedented reductions in body weight [33].

### 3.2. Drug Classes and Their Therapeutic Indications

Currently, the approved indications for GLP-1RAs include the treatment of T2D and obesity in adults, with several agents also authorized for use in pediatrics. Beyond their metabolic effects, selected GLP-1RAs have demonstrated cardiovascular risk reduction in large outcome trials and therefore they are indicated for cardiovascular risk modification in patients with established cardiovascular disease (CVD) [34].

At present, six GLP-1 based agents are available on the market: exenatide, lixisenatide, dulaglutide, liraglutide, semaglutide, and tirzepatide. Among these, exenatide and lixisenatide are synthetic analogues of exendin-4, a naturally occurring peptide originally identified in the saliva of the Gila monster, with approximately 53% sequence homology with human GLP-1 [34]. The remaining: liraglutide, semaglutide, dulaglutide and tirzepatide were developed directly based on the human GLP-1, with specific structural modifications introduced to enhance molecular stability, prolong half-life, and optimize receptor affinity [30]. These agents differ in their molecular structure resulting in clinically relevant variations in pharmacokinetics, dosing frequency (ranging from once daily to once weekly), and therapeutic efficacy, particularly with respect to weight reduction and glycemic control [31]. Most GLP-1RAs are administered via subcutaneous injection; however, oral semaglutide represents a notable exception, being the only orally available GLP-1RA, enabled by a specialized absorption-enhancing formulation [15]. The most recent addition to this therapeutic class is tirzepatide, which differs from traditional GLP-1RAs. Tirzepatide is a dual agonist of both the GLP-1 and GIP) receptors, enabling more potent glucose-lowering and weight-reducing effects compared with selective GLP-1RAs [32].

The GLP-1 therapeutic landscape continues to evolve rapidly. Ongoing research is focused on the development of novel dual and triple agonists, including agents that additionally target the glucagon receptor, as well as next-generation oral GLP-1 formulations [35].

### 3.3. Mechanism of Action and Therapeutic Potential in Addiction Treatment

GLP-1 analogs mimic the endogenous hormones secreted in response to nutrient intake, mainly by intestinal L-cells, but also pancreatic α-cells and nerve-cells in solitary tract [36]. The molecule binds to the GLP-1R, a specific receptor located in many vital organs. Through this signaling GLP-1RAs coordinate metabolic responses that extend beyond its classical pancreatic glucose regulation [17,37]. GLP-1RAs exert dual regulatory effects in the pancreas. Activation of GLP-1R on β-cells triggers cyclic adenosine monophosphate (cAMP) dependent signaling pathways that enhance glucose-stimulated insulin secretion, strengthening both, the early and sustained postprandial insulin response [38]. Chronic stimulation further promotes β-cell differentiation and survival [37]. Simultaneously, GLP-1 analogs suppress glucagon secretion. In α-cells, GLP-1RA activates a similar cAMP-induced pathway; however, this mechanism inhibits glucagon release [39]. Likewise, δ-cells are stimulated to secrete somatostatin-a hormone that promotes insulin release and suppresses glucagon [40,41]. Importantly, GLP-1-mechanism loses its activity at low plasma glucose levels, preventing dangerous hypoglycemia [42].

In the gastrointestinal system, GLP-1 analogs slow gastric emptying and diminish motility [43]. The molecule binds to its receptors on vagal afferent fibers, enhancing signaling to the brainstem. This vagal activation stimulates central pathways in the nucleus tractus solitarius that reduce efferent vagal tone to the stomach [44,45]. As a result, gastric smooth-muscle contractility decreases, delaying nutrient absorption and blunting postprandial glucose excursions [17]. Hepatic effects of GLP-1RAs occur indirectly through increased adiponectin secretion, promoting hepatic insulin sensitivity and attenuating gluconeogenesis. GLP-1 signaling also contributes to reductions in liver fat content, inflammation, and oxidative stress, making it beneficial in metabolic-associated liver disease [17,46]. Moreover, GLP-1RAs affect adipose tissue via the sympathetic nervous system. It enhances norepinephrine release, which drives lipolysis, triglycerides catabolism and fatty-acid oxidation, while also modulating the browning of white adipose tissue [47,48]. Another mechanism of action include food intake regulation. GLP-1 analogs target the arcuate nucleus in the hypothalamus, responsible for regulating orexigenic and anorexigenic signaling pathways, contributing to the normalization of body fat mass and body weight setpoint [37]. Beyond their metabolic effects, GLP-1RAs exert direct cardiovascular benefits, including reductions in atherosclerotic events. Moreover, they appear to improve endothelial function and reduce inflammation, platelet aggregation, and thrombogenicity. GLP-1 analogs lower blood pressure through natriuretic effect [34,49,50]. In kidneys they provide renal protection, by reducing albuminuria, slowing eGFR decline, and improving renal vascular function [31,51]. While the metabolic and cardiovascular benefits of GLP-1RAs are well established in clinical settings, the underlying central nervous system mechanisms, particularly those involving reward processing, remain under active investigation.

**Figure 1:**
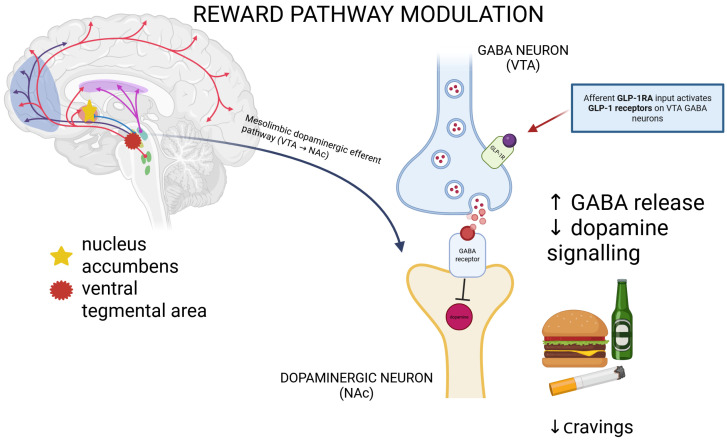
Proposed mechanism of inhibition of the dopaminergic pathway by GLP-1. GLP-1 receptor activation modulates mesolimbic reward circuitry. GLP-1 receptor agonists provide afferent input by acting on GLP-1 receptors expressed on GABAergic neurons within key reward-related regions, including the ventral tegmental area (VTA). Activation of these receptors enhances GABA release onto dopaminergic neurons in the VTA, resulting in reduced dopaminergic efferent signaling along the mesolimbic pathway from the VTA to the nucleus accumbens (NAc). This suppression of dopaminergic signaling is thought to attenuate reward salience and motivational drive associated with food cues and a broad range of addictive substances, including drugs, alcohol, and nicotine, ultimately resulting in reduced craving and substance-seeking behavior [53,55]. Increase ↑; decreased ↓.

Neurologically, GLP-1 signaling demonstrates neuroprotective actions, including reduced neuroinflammation, improved cerebrovascular function, and potential benefits in stroke recovery and neurodegenerative diseases [37,52]. Moreover, GLP-1RAs appear to influence reward circuitry within the mesolimbic system, particularly the ventral tegmental area and nucleus accumbens. By modulating GABAergic and glutamatergic transmission onto dopaminergic neurons, they are thought to attenuate reward-driven behaviors, diminishing cravings not only for food but also for substances such as alcohol, nicotine, and cocaine. While dopaminergic modulation is considered a central component of GLP-1-mediated effects on reward processing, evidence suggests that additional non-dopaminergic mechanisms, including glutamatergic signaling and brainstem inputs, may also contribute [53,54]. The mechanism of inhibition of the dopaminergic pathway is presented in Figure 1.

## 4. Incretins and Alcohol

WHO estimates that approximately 280 million people suffer from alcohol use disorders (AUD) [56]. AUD are associated with high mortality rates due to medical complications, injuries [57], and suicide [58]. AUD pose serious consequences not only for the individual with the condition but also for the family and society due to high healthcare and socioeconomic costs [54]. Harmful alcohol use is estimated to cause over 5% of deaths worldwide, making it the leading cause of preventable death [56]. AUD is a chronic, relapsing brain disorder characterized by loss of control over alcohol consumption, compulsive drinking behavior leading to relapse, and a negative affective state when not drinking [57]. Up to 50% of AUD patients experience alcohol withdrawal symptoms such as nausea, tremors, and anxiety, and some require medical assistance for detoxification [59]. Several behavioral and psychological therapies for AUD are available and have been shown to be effective in clinical trials [60]. The European Medicines Agency (EMA) has approved four pharmacological treatments, namely disulfiram, acamprosate, naltrexone, and nalmefene, whereas the US FDA has approved three treatments- disulfiram, acamprosate, and naltrexone [61]. However, despite the availability of pharmacotherapy, success rates in achieving long-term abstinence are low, underscoring the urgent need for new and more effective medications to treat AUD. In the search for new treatments, GLP-1 has attracted considerable interest [54]. Growing evidence suggests that the GLP-1 signaling is involved in the neurobiology of addictive behaviors. It modulates alcohol-related behaviors, particularly through its actions on neural circuits responsible for reward and appetite control. Therefore, it has been hypothesized that GLP-1 analogs could be used to treat AUD [55].

### 4.1. Preclinical Study

Several GLP-1RAs have been evaluated in preclinical models of addiction for their effects on alcohol consumption in rodents and non-human primates. Preclinical studies in the conditioned place preference (CPP) model demonstrate a reduction or abolition of alcohol place preference when animals are pretreated with systemic exenatide or when exenatide is administered centrally to the nucleus tractus solitarius (NTS) or NAc. Similar results were obtained with liraglutide [59]. Studies have shown promising results for liraglutide in reducing alcohol consumption, self-administration, and alcohol preference in rats [62,63]. EX-4 has been shown to inhibit alcohol-induced locomotion, NAc dopamine release, voluntary alcohol consumption, and seeking behavior in mice [64]. Furthermore, lower intravenous alcohol self-administration was observed in males treated with EX-4 [65]. Systemic injection of EX-4 not only reduced alcohol consumption but also altered drinking patterns. EX-4 reduced the number of drinking episodes and increased the time to the first drinking episode [66]. Local administration of EX-4 to the ventral tegmental area (VTA) prevented the ability of ghrelin, a well-known orexigenic peptide, to increase alcohol consumption in an intermittent access model as well as an operant alcohol-responsive model [67]. EX-4 dose-dependently reduced the urge to consume alcohol in male rats when tested in an operant self-administration model [64,68]. Systemic administration of EX-4 reduced operant alcohol self-administration in male mice. The efficacy of EX-4 in reducing the motivation to consume alcohol was less pronounced in female mice [69]. Alcohol seeking in the progressive ration test was attenuated by systemic injection of EX-4 in rats [64] and male mice, whereas there was no effect on alcohol-seeking female mice [69]. Studies with semaglutide have shown dose-dependent reductions in alcohol consumption in both addicted and non-addicted mice and rats of both sexes [55,70]. Marty et al. demonstrated lower alcohol consumption in male rats acutely treated with semaglutide than in those treated with vehicle [63]. Subsequently, acute and repeated injections of semaglutide dose-dependently reduced alcohol consumption and alcohol preference in both male and female rats, with the effect being stronger in females [70,71]. Chuong et al. demonstrated that semaglutide reduced binge drinking in mice and rats [55]. Fink-Jensen et al. reported that semaglutide administration reduced alcohol consumption in alcohol-preferring male vervet monkeys [72]. Dulaglutide treatment reduced alcohol consumption in male and female rats, but this effect was stronger in males. Reduced alcohol consumption and preference were evident during 5 or 9 weeks of dulaglutide treatment. The treatment effect persisted for an additional 3 weeks after treatment discontinuation [73]. Available evidence indicates that research has identified significant sex differences in the effects of GLP-1RAs in relation to alcohol use. The mechanisms underlying the observed sex differences in response to GLP-1RAs are not yet fully understood. Potential mechanisms may include modulation of GLP-1 receptor signaling by sex hormones, sex-specific differences in pharmacokinetics, and variability in reward-related neural circuits. These factors may contribute to the observed behavioral differences, emphasizing the need to consider sex as a biological variable in preclinical and clinical studies of GLP-1RAs in addiction [74]. A significant contributor to the complexity of AUD is relapse to drinking, which is commonly observed after periods of abstinence. In animal models, this can be studied using an alcohol deprivation model, in which alcohol is deprived before rechallenge. Using this model, acute systemic injection of EX-4 reduced the odds of relapse to drinking in male rats [62] as well as in male mice housed in social groups [66]. Similarly, liraglutide [75] and semaglutide [70] inhibited relapse to drinking in male mice. During withdrawal, both mice and rats exhibit withdrawal symptoms, which are alleviated by systemic injection of liraglutide [75,76]. Although motivation to drink alcohol and relapse have not been studied in relation to central GLP-1, increasing peripheral GLP-1 levels (by a dipeptidyl peptidase-4 (DPP-IV) inhibitor) has been found to reduce withdrawal symptoms [77].

### 4.2. Human Studies

Several studies have been published on the influence of GLP-1RAs on alcohol consumption in humans. Initial findings demonstrated an association between GLP-1RA and alcohol in a small, preliminary pilot study of T2D patients, who self-reported reduced alcohol consumption after treatment with liraglutide [78]. Subsequently, Suchankova et al. conducted a human genetic study demonstrating an association between single-nucleotide polymorphisms (SNPs) of the *GLP-1R* gene and the diagnosis of AUD in two separate cohorts of normal-weight AUD patients. In this study, a positive correlation was observed between SNPs of the *GLP-1R* and intravenous alcohol self-administration and breath alcohol levels in alcohol users [79]. Farokhnia et al. subsequently confirmed these human genetic findings in a normal-weight population of individuals at low and high risk of alcohol consumption. An association has been noted between *GLP-1R* polymorphisms and Alcohol Use Disorder Identification Test (AUDIT) scores [80]. A randomized clinical trial (RCT) demonstrated an interaction between GLP-1 and alcohol, as daily injections of exenatide reduced alcohol consumption in overweight patients with AUD. This association was not observed in normal-weight individuals [81]. Probst et al., in another RCT, assessed the efficacy of dulaglutide in reducing alcohol consumption among smoking patients with AUD, whose body weight varied but who were predominantly obese. Alcohol consumption decrease was observed after 12 weeks of dulaglutide treatment, and this effect was achieved regardless of smoking status, initial alcohol consumption, or body weight [82]. RCT carried out by Hendershot et al. demonstrated that low-dose semaglutide significantly reduced alcohol consumption during a post-treatment laboratory self-administration procedure compared with placebo. Over 9 weeks of treatment, low-dose semaglutide reduced alcohol consumption during post-treatment self-administration in the laboratory, demonstrating a medium to large effect size for grams of alcohol consumed and peak breath alcohol concentration. Semaglutide treatment did not affect number of average drinks per calendar day or number of drinking days, but significantly reduced the number of drinks per drinking day and weekly craving, which also predicted a greater reduction in heavy drinking over time compared with placebo (*p* = 0.04) [83]. Additionally, in a small sample of patients with overweight and comorbid AUD, semaglutide reduced AUDIT scores [84].

Retrospective cohort analyses showed consistent and promising results regarding the associations between GLP-1RAs and alcohol-related adverse health outcomes. An interaction between GLP-1RA and alcohol was found in a Danish nationwide cohort study examining the risk of alcohol-related events in patients prescribed GLP-1-based therapies [85]. This registry-based study showed that alcohol-related events were reduced in patients prescribed with GLP-1RA when compared to individuals treated with DPP-IV inhibitors [85]. Wang et al. conducted a retrospective cohort study using data from electronic medical records that examined the associations between semaglutide and incidence or recurrence of AUD. The authors stratified analyses by obesity and T2D diagnoses, comparing semaglutide with the AUD medications naltrexone and topiramate, as well as other non-GLP-1 obesity and diabetes medications. Among patients with obesity, those taking semaglutide had a 56% reduced risk of AUD recurrence (compared to non-GLP-1RA anti-obesity medications) and a 75% reduced risk of AUD recurrence compared with those prescribed naltrexone or topiramate for a 12-month follow-up period. The study demonstrated protection against incident and recurrent AUD diagnoses in individuals with obesity compared with non-GLP-1 obesity medications, as well as in individuals with diabetes compared with non-GLP-1 diabetes medications. The study also demonstrated that the protective effect persisted in the subgroup of those taking the medications for three years [86]. Qeadana et al., in a retrospective cohort analysis, used medical record data from more than 800,000 adults with AUD from 136 US health systems. In adjusted models, GIP and/or GLP-1RA use was associated with a 50% reduction in the incidence of alcohol intoxication [87]. Similarly to the study by Wang et al., these results remained consistent in analyses stratified by diabetes and obesity diagnoses [86]. Lähteenvuo et al. conducted an analysis using the full census of Swedish adults with AUD. They found that semaglutide and liraglutide were associated with a 36% and 28% reduction in the risk of AUD-related hospitalization over time. In contrast, use of any AUD medication (i.e., naltrexone, acamprosate, disulfiram) was associated with only a 2% reduction in the risk of AUD-related hospitalization. Use of semaglutide and liraglutide was associated with a reduced risk of hospitalization for substance use disorders and physical illnesses. This study also examined the association between exenatide and hospitalizations. The results were not statistically significant; however, it should be noted that the number of exenatide user was relatively small [88]. Bremmer et al. conducted a social media study analyzing anonymous online reports of patients on GLP-1 therapy. They found that individuals on GLP-1RA therapy self-reported lower rates of alcohol cravings, interest in alcohol, and alcohol consumption [89]. Arillotta et al. explored user-reported experiences with GLP-1 RAs in relation to substance use, compulsive behaviors, and libido using social media data. The findings indicated that a substantial proportion of users reported a reduction or complete cessation of alcohol consumption following initiation of GLP-1 RA therapy, with nearly 30% of alcohol-related comments describing decreased intake. Similar trends were observed for nicotine and caffeine use, suggesting a possible attenuation of substance-related cravings. Additionally, approximately one-fifth of comments referring to compulsive shopping described a perceived reduction in impulsive purchasing behaviors. The study suggests that GLP-1 RAs may influence reward-related behaviors and substance use, potentially through central nervous system pathways involved in appetite and reward regulation. Nevertheless, the authors emphasize that these findings are exploratory and hypothesis-generating, as the data are derived from self-selected social media users and do not allow for causal inference [90]. Machine learning analysis of social media posts showed that users associate GLP-1 medications lower with cravings, reduced usage and other negative effects of drinking. Furthermore, in the remote study, individuals with obesity taking semaglutide or tirzepatide reported significantly lower self-reported alcohol intake, fewer binge drinking episodes, and reduced AUDIT scores compared with both baseline measurements prior to treatment and the control group. The observed reductions in alcohol consumption in these patients with obesity may be related to the ability of semaglutide and tirzepatid to reduce the stimulating and sedative effects of alcohol [91]. Meta-analysis performed by De Faria Moraes et al., outlines that the evidence from the observational studies is promising, demonstrating an association between GLP-1RAs use and fewer alcohol-related events and liver-related outcomes. However, these encouraging observational findings must be interpreted with caution due to their inherent potential for confounding and bias. Variations in baseline characteristics such as age, sex, BMI, and metabolic status may affect treatment responses. Comorbid conditions, including psychiatric disorders or other substance use disorders, can also modulate outcomes. Additionally, lifestyle behaviors (e.g., diet, physical activity, smoking) and concomitant medications may influence both the efficacy and observed effects of GLP-1RAs. These factors can complicate causal inference and limit the generalizability of results. While observational data provide valuable insights, these limitations should be taken into account when extrapolating results and drawing conclusions about causality. Moreover, data from RCTs remain limited, as the pooled estimate for RCTs did not find a statistically significant association [92]. Despite the fact that reduction in alcohol consumption is observed after GLP-1R activation, the specific neural circuits and mechanisms underlying this effect remain to be defined in humans, as in animal models. While most of these studies report that GLP-1RAs significantly reduce alcohol consumption in patients with excessive weight, the results in individuals with normal weight are understudied. This is understandable, as this class of medications is currently approved primarily for the treatment of T2D and obesity; and consequently, most available RCTs have been conducted in populations with those conditions. However, given the potential effects of these medications on reward-related behaviors, future RCTs should directly compare their impact on alcohol consumption in individuals with normal body weight versus those with obesity. Additionally, further research is needed to clarify whether, and to what extent, these medications influence body weight in individuals with normal baseline body mass index (BMI), as existing evidence in this population remains limited. It is also important to note that there are some discrepancies between the preclinical and clinical studies conducted to date, as they have studied normal-weight animals and primarily humans with overweight. Preclinical and clinical studies are still in their early stages, and firm conclusions regarding which patient groups may respond best remain unclear.

Further research is needed to understand how these medications might work in combination with existing medications for AUDs, as well as with psychosocial interventions that could address the complex social and mental health needs common among individuals with AUDs. Table 1 provides an overview of currently ongoing clinical trials investigating the effects of GLP-1RA on AUD. Results from those studies may provide answers to the remaining scientific questions regarding use of those medications in AUD treatment.

## 5. Incretins and Nicotine Use

Tobacco use represents a significant global health burden, accounting for over 7.7 million deaths and 200 million disability-adjusted life-years annually [93,94,95]. Despite the majority of smokers expressing a desire to quit, long-term abstinence rates remains low. Fewer than 10% achieve sustained cessation with existing pharmacotherapies, and unassisted quit attempts fail in 95-98% of cases [54,96].

Several pharmacological interventions are available to support smoking cessation, including nicotine replacement therapy (NRT), varenicline, and bupropion. Among these, NRT is the most commonly prescribed and demonstrates a favorable safety profile across diverse populations [97]. Clinical trials indicate that NRT increases quit rates by approximately 50% relative to no pharmacotherapy. Nevertheless, relapse remains common, long-term outcomes are variable, and combination therapies tend to achieve higher abstinence rates than monotherapy, highlighting the limitations of single-agent interventions [97].

Beyond their metabolic effects, GLP-1 RAs exert significant actions in the central nervous system relevant to addiction [14,30,98,99]. GLP-1R activation modulates mesolimbic dopaminergic pathways by inhibiting nicotine-evoked dopamine release in the NAc, thereby reducing nicotine’s reinforcing properties [14,100]. Early preclinical evidence supporting these mechanisms was provided by Egecioglu et al. In male NMRI mice, systemic administration of Ex-4 (2.4 μg/kg, intraperitoneally) prior to nicotine exposure (0.5 mg/kg, i.p.) significantly attenuated nicotine-induced locomotor stimulation and blocked nicotine-evoked increases in extracellular dopamine in the NAc. Ex-4 did not affect baseline locomotion, indicating a stimulus-specific neuromodulatory effect. Furthermore, a single Ex-4 injection abolished nicotine-induced conditioned place preference, while repeated administration prevented the development of locomotor sensitization. Together, these findings support the hypothesis that GLP-1R activation reduces nicotine reward without inducing aversive effects [101]. Herman et al. extended these observations by investigating liraglutide in both male and female rats trained to self-administer intravenous nicotine (0.03 mg/kg/infusion). Daily liraglutide treatment (25 μg/kg, i.p., daily) substantially attenuated nicotine-seeking behavior during cue- and nicotine-induced reinstatement, indicating diminished relapse vulnerability to drug-primed or conditioned stimuli. Furthermore, repeated liraglutide administration alleviated withdrawal-induced hyperphagia and weight gain—frequent relapse precipitants—without altering general food intake or motor activity, thus emphasizing its selective effects [102].

In addition to its modulation of mesolimbic pathways, GLP-1 signaling recruits habenular aversion circuits, acting as a homeostatic satiety sensor for nicotine [103]. Nicotine stimulates GLP-1 neurons in the NTS which project to the medial habenula (MHb)—interpeduncular nucleus (IPN) pathway and potentiate excitatory glutamatergic transmission from the MHb to the IPN. Activation of GLP-1Rs in this circuit attenuates nicotine reward, elicits avoidance, and curbs intake, whereas disruption of the pathway elevates consumption [14,100,103]. Tuesta et al. provided mechanistic validation of this pathway. Employing chemogenetic, optogenetic, and pharmacological techniques, they demonstrated that augmenting GLP-1 signaling through Ex-4 or the DPP-4 inhibitor sitagliptin decreased nicotine intake, while GLP-1R knockout or circuit-specific antagonism in the MHb–IPN pathway elevated consumption. Optogenetic activation of GLP-1 projections to this circuit eliminated nicotine reward without altering food intake, underscoring the pathway’s involvement in rapid avoidance mechanisms and delineating a neuroanatomical connection between the gut–brain axis and habenular circuits in nicotine-related behavior [103].

Building upon these preclinical findings, investigations have extended to human populations to assess the effects of incretin-based therapies. Arillotta et al. conducted a mixed-methods analysis of self-reported modifications in addictive behaviors among users of GLP-1RAs. The investigation primarily utilized social media data, focusing on Reddit posts gathered from December 2019 to June 2023. The aim was to assess the perceived impacts of GLP-1RAs—such as semaglutide and tirzepatide—on consumption of psychoactive substances (e.g., nicotine). Results indicated that incretin-based therapy was linked to reductions in both substance use and behavioral addictions, including lower nicotine intake. Nevertheless, the authors highlighted limitations inherent to self-reported data, including the lack of standardized assessments and susceptibility to reporting biases, which prevent causal attributions; thus, these preliminary insights necessitate validation through prospective clinical trials [90]. Subsequent pilot RCTs have assessed the efficacy of GLP-1 RAs in promoting smoking cessation. Yammine et al. randomized 84 prediabetic and/or overweight smokers to extended-release exenatide combined with nicotine replacement therapy patches and behavioral counseling or a placebo regimen for 6 weeks. The active intervention significantly boosted end-of-treatment abstinence rates to 46.3% in the exenatide arm versus 26.8% in placebo, while also alleviating withdrawal symptoms and cravings in abstainers and curbing post-cessation weight gain by approximately 5.6 pounds. These results indicate that GLP-1RA therapy holds promise for improving cessation outcomes and mitigating weight gain, warranting evaluation in larger, extended-duration trials [97]. However, a subsequent larger trial by Lüthi et al. yielded more tempered findings. This single-center, randomized, double-blind, placebo-controlled study assessed dulaglutide as an adjunct to standard smoking cessation interventions, enrolling 255 participants (randomized 127 to dulaglutide and 128 to placebo). Dulaglutide recipients initiated subcutaneous injections at 0.75 mg in week 1, titrating to 1.5 mg weekly thereafter for 11 weeks, whereas the placebo arm received 0.5 mL of 0.9% NaCl weekly. Both groups underwent concomitant standard care with varenicline and behavioral counseling. Following the 12-week treatment phase, abstinence rates were equivalent between arms. At 52-week follow-up, sustained abstinence was observed in 32% of participants in each group. Although dulaglutide conferred no long-term cessation advantage, it initially prevented post-cessation weight gain during treatment. Median weight changes at 12 weeks comprised −1.0 kg versus +1.9 kg; by week 24, values rose to +1.5 kg and +3.0 kg, respectively. By week 52, median gains equilibrated at +2.8 kg and +3.1 kg (compared to baseline). Collectively, these findings suggest that dulaglutide does not improve long-term smoking cessation, but may have a potential to prevent weight gain in the first months after quitting. The lack of efficacy for nicotine dependence may be associated with short treatment duration and agent-specific pharmacological properties, including limited central nervous penetration. Moreover, the authors described several methodological limitations, such us missing data and sample size estimation [104].

Taken together, preclinical and initial clinical data reveal that GLP-1R signaling influences nicotine-linked behaviors by engaging both mesolimbic reward circuitry and habenular aversion mechanisms. Animal investigations consistently report pronounced attenuations in nicotine reinforcement, reinstatement tendencies, and withdrawal-induced weight gain, in contrast to the circumscribed and disparate human data. Existing clinical studies posit that GLP-1RAs offer metabolic advantages associated with weight gain prevention during tobacco withdrawal, albeit exerting merely modest or equivocal impacts on sustained abstinence. Consequently, larger, well-powered RCTs are warranted to clarify if different GLP-1RAs (and their multi-agonists) should be considered as effective adjunctive agents in nicotine dependence therapy.

## 6. Incretins and Psychoactive Substances

Reward-related behaviors in substance use disorders involve mesocorticolimbic circuits, particularly the VTA, NAc, amygdala, orbitofrontal cortex (OFC), anterior cingulate cortex (ACC), and prefrontal cortex (PFC), where dopaminergic signaling drives reinforcement learning [105]. Drug exposure paired with environmental cues strengthens synaptic connections, consolidating associations between drug-seeking actions and reward, which promotes craving and relapse [106]. Similar circuits are activated by food cues in obesity, highlighting overlapping mechanisms of motivated behavior [107,108,109]. Given that both conditions rely on dysregulated mesocorticolimbic signaling, it is reasonable to consider whether treatments that normalize food-cue reactivity, such as GIP/GLP-1RAs, might exert similar regulatory effects on drug-cue reactivity.

### 6.1. Psychostimulants

Psychostimulants such as cocaine, amphetamine, and methamphetamine exert their addictive properties by enhancing dopaminergic and noradrenergic signaling within the mesolimbic reward pathway through partially distinct mechanisms [110]. Cocaine primarily increases extracellular dopamine by blocking the dopamine transporter (DAT), whereas amphetamine and methamphetamine reverse DAT function, promoting calcium-independent dopamine release [111]. Despite these mechanistic differences, both drug classes produce rapid elevations in dopamine levels, particularly in the NAc. Rapidity of this dopaminergic surge is a critical determinant of euphoria intensity and addiction liability [111].

Against this neurobiological backdrop, growing preclinical evidence indicates that GLP-1RAs effectively counteract stimulant-induced behavioral and neurochemical alterations. Across multiple rodent models, GLP-1RAs were shown to reduce cocaine- and amphetamine-induced hyperlocomotion, suppress cocaine self-administration, attenuate accumbal dopamine release, and decrease CPP as well as reinstatement behaviors. Collectively, these findings suggest a capacity of GLP-1RAs to blunt both drug reward and relapse-like responding [112,113,114]. These effects appear to be mediated by GLP-1R activation within key mesolimbic structures, particularly the VTA and the NAc, where stimulation of GLP-1Rs dampens cocaine-evoked dopamine elevations and reduces the reinforcing efficacy of stimulants. This modulation likely occurs through suppression of dopaminergic neuron activity and enhancement of local inhibitory GABAergic signalling [115,116]. Hernandez et al. demonstrated that Ex-4 reduces cocaine-seeking behavior by activating GLP-1Rs, which are predominantly expressed on GABAergic neurons in the laterodorsal tegmental nucleus (LDTg). This effect was mediated, at least in part, via GABAergic LDTg projections to the VTA, revealing a critical role for LDTg GLP-1Rs in regulating relapse to addictive behaviors [117]. In line with these findings, Ex-4 readily crosses the blood–brain barrier and interacts with GLP-1Rs expressed on both neurons and astrocytes within the VTA [118]. More recent work further supports this inhibitory framework, showing that systemic administration of GLP-1RAs suppresses cocaine-seeking behavior by enhancing the activity of VTA GABAergic neurons, thereby reducing dopaminergic neuron firing [116].

Beyond the VTA, GLP-1R signaling within the NAc also plays a distinct role in regulating relapse vulnerability. Local activation of GLP-1Rs in the NAc by Ex-4 reduced cocaine-primed reinstatement following extinction and selectively increased the intrinsic, but not synaptic, excitability of medium spiny neurons in cocaine-experienced rats [119]. These findings suggest that enhanced NAc GLP-1R signaling during abstinence is sufficient to suppress cocaine-seeking behavior primarily through modulation of neuronal excitability rather than synaptic transmission. Consistent with this interpretation, Zhu et al. reported that GLP-1RAs reduce both cocaine- and stress-induced reinstatement of CPP while normalizing cocaine-induced increases in nuclear factor κB (NF-κB) expression in the NAc [120].

Importantly, GLP-1R-mediated regulation of stimulant responses extends beyond classical mesolimbic structures. GLP-1Rs are highly expressed in the lateral septum (LS), a forebrain region receiving dense dopaminergic input from the VTA and strongly dependent on DAT-mediated dopamine signaling. Reddy et al. demonstrated that Ex-4 prevents cocaine-induced dopamine elevations in the LS by increasing DAT surface expression and function, partly via arachidonic acid–dependent mechanisms [121]. Finally, these convergent findings have been strengthened by recent evidence showing that semaglutide, a long-acting GLP-1RA with higher potency and receptor affinity than Ex-4, robustly reduces cocaine self-administration, motivation to consume cocaine, and reinstatement in rats, while also attenuating cocaine-evoked dopamine release in the NAc without inducing malaise [122].

Furthermore, evidence suggests that chronic cocaine exposure activates Toll-like receptor 4 (TLR4)-mediated neuroinflammatory signaling, particularly within the hippocampus [123]. As this structure is involved in associative learning and memory processes relevant for addiction, this TLR4-driven microglial activation promotes the release of pro-inflammatory cytokines, including tumor necrosis factor (TNF)-α, and interleukin (IL)-1β, which are thought to facilitate the consolidation of drug-cue associations, impair extinction learning, and enhance relapse vulnerability [123]. However, preclinical evidence indicates that Ex-4 may counteract psychostimulant-induced behaviors through anti-inflammatory mechanisms, including suppression of TLR4-dependent neuroimmune signaling and reduction in pro-inflammatory cytokines [124]. These anti-inflammatory mechanisms may contribute to attenuated reward learning, enhanced extinction, and reduced relapse propensity.

Taken together, these studies indicate that GLP-1R signaling modulates stimulant-related behaviors across multiple mesolimbic and forebrain nodes, including the VTA, NAc, LDTg, and LS. Through coordinated suppression of dopaminergic activity, enhancement of inhibitory GABAergic control, and regulation of DAT-dependent dopamine clearance, GLP-1RAs ultimately reduce cocaine intake and relapse-like responding, highlighting their therapeutic potential in the treatment of stimulant use disorders. Human data yet still remain very limited and inconclusive. Early clinical observations suggest that GLP-1 RAs may be feasible, well tolerated, and acceptable to individuals with cocaine use disorder, with preliminary signals of potential clinical benefit during several weeks of treatment [125]. However, controlled human laboratory work using acute, low-dose Ex-4 pretreatment failed to demonstrate reductions in cocaine self-administration or subjective drug effects [126]. These findings indicate that while GLP-1RAs represent a biologically plausible and promising therapeutic avenue, current human evidence is insufficient, highlighting the need for adequately powered RCTs, longer treatment durations, and evaluation of optimized dosing strategies.

### 6.2. Opioids

Opioids such as morphine, heroin, fentanyl, and oxycodone produce euphoria and reinforce drug-taking behavior primarily through activation of μ-opioid receptors on inhibitory neurons, which disinhibits mesolimbic dopamine pathways and increases dopamine release in the NAc and other reward-related regions [127,128]. Although the initial molecular targets differ from those of psychostimulants, opioids ultimately converge on the same dopaminergic circuitry that underlies reward learning, motivation, and relapse.

Within this shared neurobiological framework, accumulating preclinical evidence indicates that GLP-1RAs robustly attenuate opioid taking and seeking across multiple opioid classes. In rats, systemic administration of Ex-4 decreased oxycodone self-administration and reinstatement of oxycodone seeking without altering food intake or interfering with analgesic efficacy [129]. Mechanistically, Ex-4 crossed the blood–brain barrier and engaged GLP-1Rs expressed on both D1- and D2-receptor–positive medium spiny neurons in the NAc shell. Importantly, intra-accumbens Ex-4 infusions were sufficient to reproduce the systemic anti-opioid effects, underscoring the NAc as a key site of action [129].

Consistent effects have been observed with other GLP-1 analogs and opioid classes. Liraglutide markedly reduced multiple heroin addiction–like behaviors, including latency to initiate drug taking, total heroin intake, escalation of use, and drug-induced reinstatement, at doses that did not produce malaise or clinically relevant metabolic side effects [130]. Acute liraglutide administration (0.3 mg/kg) further suppressed heroin seeking triggered by cues, stress, and heroin priming without impairing locomotor activity, supporting the notion that GLP-1RAs may function as effective non-opioid adjuncts during early abstinence [131]. In parallel, Ex-4 was shown to reduce both cue-induced and drug-induced reinstatement of heroin seeking in a reward-devaluation model, while simultaneously increasing responsiveness to natural reward cues and modulating orexin-related signaling within the NAc shell [132].

More recent studies have extended these findings to high-potency synthetic opioids. Systemic Ex-4 reduced fentanyl self-administration and reinstatement; however, these effects were observed at doses associated with malaise-like responses. Addressing this limitation, a novel dual agonist targeting both GLP-1/neuropeptide Y2 receptors (Y2R)—GEP44, suppressed fentanyl taking and seeking without inducing nausea or reducing food intake, highlighting a potentially more tolerable, next-generation pharmacological strategy for opioid use disorders [133]. Collectively, these data identify GLP-1R signaling and possibly combined GLP-1R/Y2R activation as a key modulator of fentanyl reinforcement and relapse vulnerability.

At the circuit level, opioid seeking engages broader corticostriatal networks beyond the NAc. Functional neuroimaging studies demonstrated that inactivation of the dorsomedial striatum (DMS) reduced relapse to oxycodone seeking and altered large-scale functional connectivity, implicating corticostriatal control systems in opioid relapse [134]. These findings point to potential interaction sites with GLP-1–mediated signaling pathways that regulate motivation, action selection, and relapse propensity. Notably, however, the literature is not entirely consistent. One study reported that Ex-4 failed to reduce morphine-induced CPP, withdrawal responses, or remifentanil self-administration in mice, despite using doses that were behaviorally active and sufficient to suppress alcohol self-administration under identical experimental conditions [68]. These results suggest that GLP-1–based modulation of drug-seeking behavior may differ across opioid classes and experimental paradigms. Importantly, the lack of efficacy cannot be readily attributed to insufficient dosing or nonspecific behavioral effects, but rather points to opioid-specific mechanisms of reinforcement and dependence that may be less sensitive to GLP-1–mediated modulation. As suggested by the authors, full μ-opioid receptor agonists such as morphine and remifentanil may engage reinforcement-related circuitry that is less responsive to GLP-1R signaling, or may require alternative dosing strategies or chronic treatment paradigms to reveal behavioral effects [68]. This divergence is not easily explained by methodological variables and may instead reflect fundamental differences in how GLP-1R signaling interacts with reward circuitry across drug classes.

Importantly, emerging clinical data provide early support for the translational relevance of these preclinical findings. Treatment with semaglutide in patients with T2D and comorbid opioid use disorder was associated with a reduced risk of opioid overdose, suggesting a potential protective effect in humans [135]. In line with this observation, a large retrospective cohort study using de-identified electronic health records from the Oracle Cerner Real-World Data platform reported that treatment with GIP and/or GLP-1RAs was associated with a lower incidence of opioid overdose among individuals diagnosed with opioid use disorder [87]. Building on these observational signals, clinical translation is actively underway. Two randomized, placebo-controlled trials are currently evaluating liraglutide in residential treatment settings and semaglutide in outpatient medication-assisted treatment programs, focusing on abstinence, craving, and opioid use during ongoing methadone or buprenorphine therapy [136,137]. Together, these studies aim to determine whether the robust preclinical effects of GLP-1–based therapies translate into meaningful clinical benefit for opioid use disorder.

### 6.3. Cannabinoids

Although GLP-1RAs have not yet been systematically evaluated in experimental models of cannabis or Δ9-tetrahydrocannabinol (THC) use disorders, emerging evidence points to several mechanistic intersections between the endocannabinoid and GLP-1 systems that may be relevant to cannabis use disorder (CUD). Cannabinoid type 1 receptors (CB1) are widely expressed within stress- and reward-related circuits, including the lateral habenula, VTA, and bed nucleus of the stria terminalis (BNST), where they regulate anxiety-like behavior, modulate GABAergic control over dopaminergic firing in the VTA, and shape behavioral responses to drug-associated cues [138,139,140]. Notably, GLP-1Rs are also expressed in several of these regions, including the BNST, where chronic suppression of GLP-1Rs has been shown to reduce anxiety-like behavior [141]. This anatomical and functional overlap suggests that CB1–GLP-1 interactions within stress- and reward-related circuits may represent a plausible interface through which GLP-1R activation could influence cannabis-related behaviors.

At the molecular level, bidirectional modulation between the two systems further supports this possibility. Endocannabinoid-related lipids such as oleoylethanolamide (OEA) and 2-oleoylglycerol (2-OG) have been shown to enhance GLP-1–induced cAMP signaling, whereas CB1 activation suppresses GLP-1–mediated insulinotropic responses, indicating reciprocal regulatory control [142]. Endocannabinoids, acting through the CB1, can inhibit the action of GLP-1 in regulating insulin secretion, suggesting that the cannabinoid system may modulate mechanisms linking the control of energy metabolism and reward signaling [143].

Beyond these mechanistic considerations, emerging real-world clinical data provide the first indication that GLP-1RAs may reduce vulnerability to CUD. In a large retrospective cohort study, Wang et al. analyzed health records from over 85,000 patients with obesity and found that semaglutide treatment was associated with a substantially lower risk of both incident (hazard ratio [HR]: 0.56, 95% confidence interval [CI]: 0.42–0.75) and recurrent (HR: 0.62, 95% CI: 0.46–0.84) CUD diagnoses compared with non–GLP-1RA anti-obesity medications, with consistent effects across demographic subgroups [144]. Although observational in nature and not designed to establish causality, these findings raise the possibility that GLP-1RAs may attenuate cannabis-related dysregulation in humans.

Taken together, converging preclinical and observational evidence suggests that GLP-1RAs may influence cannabis-related behaviors through bidirectional CB1–GLP-1 interactions within stress and reward circuits. While direct experimental and clinical data remain limited, these observations provide a rationale for future mechanistic studies and controlled clinical trials evaluating GLP-1–based therapies in cannabis use disorder.

A detailed summary of the neural and molecular mechanisms through which GLP-1RAs modulate psychostimulant-related behaviors is provided in Table 2.

## 7. Clinical Perspectives and Future Research Directions

Accumulating evidence suggests that incretin-based therapies may represent a novel approach to the treatment of substance use disorders. Preclinical studies consistently demonstrate reductions in alcohol intake, nicotine consumption, and drug-seeking behaviors across multiple classes of psychoactive substances, including psychostimulants, opioids and cannabinoids. The proposed mechanisms underlying these effects include modulation of reward circuitry within the mesolimbic system, particularly in the ventral tegmental area and nucleus accumbens. By modulating GABAergic and glutamatergic transmission onto dopaminergic neurons, GLP-1RAs attenuate reward-driven behaviors and diminish cravings not only for food but also for addictive substances.

Human data are still emerging and include mostly observational studies, which further support potential clinical benefits, including reductions in substance use severity and relapse-related outcomes. Nevertheless, the currently available research still does not determine whether, and to what extent, incretin-based therapies may be a safe and effective treatment for different substance use disorders. Retrospective cohorts, small treatment groups, observational design and social media analyses are associated with various methodological limitations and are highly susceptible to confounding and reporting bias. Preclinical studies of GLP-1Ras in addiction models demonstrate considerable heterogeneity that can influence the interpretation of outcomes. Differences across species and strains, as well as sex-specific effects, have been reported, with certain behavioral responses being more pronounced in specific models. Experimental paradigms, including CPP, self-administration, and reinstatement protocols, further contribute to variability in observed effects. In addition, short-acting and long-acting GLP-1RAs differ in pharmacokinetic properties and dosing regimens, which can impact both efficacy and behavioral outcomes. Recognizing these sources of heterogeneity is critical for contextualizing preclinical findings and for informing translational research aimed at evaluating incretin-based therapies in human addiction. Therefore, the available results should be interpreted with caution.

From a clinical perspective, incretin-based agents are particularly attractive given their established safety profile and widespread metabolic benefits. While current evidence remains insufficient to support routine clinical use in addiction treatment, findings presented in this article provide a strong rationale for well-designed RCTs to determine efficacy, optimal dosing and patient subgroups most likely to benefit. A particularly important research question concerns the potential efficacy and safety of this class of medication in metabolically healthy individuals with substance use disorders. Most available studies on GLP-1RAs in addiction have been conducted in individuals with overweight or obesity, while data on participants with normal weight remain scarce. This restricts the generalizability of current findings, as pharmacokinetics, hormonal milieu, neural reward responses, and susceptibility to addiction-related behaviors may differ between normal-weight population and those with overweight/obesity. Future studies should carefully evaluate possible dosing regimens and adverse events profiles, including outcomes such as changes in body weight and hypoglycemia risk.

Moreover, it should be taken into account that incretin-based therapies represent a heterogeneous class of agents with distinct pharmacological profiles; therefore, therapeutic effects on addiction-related outcomes are likely to vary across studied medications.

## 8. Conclusions

If validated in clinical trials, incretin-based therapies could offer a promising adjunctive strategy for the management of substance use disorders, addressing both metabolic comorbidity and addiction-related neurobiology. The possibility of using multiple agonists in therapies further expands the therapeutic landscape, offering opportunities to optimize efficacy by simultaneously targeting multiple metabolic and reward-related pathways. Nevertheless, the currently available research is still insufficient to support their routine clinical use in addiction treatment.

## Figures and Tables

**Table 1:** Unpublished or ongoing clinical trials of a GLP-1 receptor agonist for the treatment of alcohol use disorder registered by 18 January 2026.

NCT Identifier	Drug	Administration	*n* =	Study Group	Comparator	Primary Outcome	Expected End Date
NCT05895643	Semaglutide	sc, 2.4 mg once weekly	108	individuals diagnosed with alcohol use disorder and comorbid obesity (BMI > 30 kg/m^2^).	placebo	Change in heavy drinking days	December 2025
NCT05891587	Semaglutide	sc, 1.0 mg once weekly	80	individuals who endorse symptoms consistent with alcohol use disorder	placebo	Change in alcohol drinking measured per week	December 2025
NCT05892432	Semaglutide	po, 7.0 mg once daily	50	treatment-seeking individuals with AUD	placebo	Change in alcohol craving	October 2025
NCT06015893	Semaglutide	sc, 2.4 mg once weekly	52	individuals with AUD	placebo	Change in alcohol consumption	December 2030
NCT07249554	Semaglutide + Naltrexone	po, 7.0 mg once daily	45	individuals with AUD	placebo + placebo, placebo + GLP-1, GLP-1 + Naltrexone	Participant-reported Adverse Events	June 2028
NCT07218354	Semaglutide	sc, 2.4 mg once weekly	438	veterans with AUD	placebo	Two-level reduction in the WHO risk drinking level	January 2029
NCT06546384	Semaglutide	sc, 2.4 mg once weekly	64	individuals with obesity and fatty liver disease	nutritional and exercise recommendations	Proportion of patients achieving total alcohol abstinence (measured by negative PEth test)	April 2027
NCT06994338	Tirzepatide	sc, 5 mg once weekly	42	individuals with AUD and overweight or obesity	placebo	Number of heavy drinking days	August 2026
NCT07221214	Semaglutide	po, 7.0 mg once daily	200	individuals living with HIV	placebo	Average drinks/week past 30 days at 3 months	July 2030
NCT06939088	Tirzepatide	sc, 15 mg once weekly	108	individuals with a dual diagnosis of AUD and schizophrenia	placebo	Change in heavy drinking days	December 2028
NCT06727331	Tirzepatide	sc, 2.5 mg once weekly	20	individuals with AUD	placebo	Cue-induced Cravings for Alcohol and Incidence and Severity of Adverse Events	July 2026
NCT07046819	Tirzepatide	sc, 7.5 mg once weekly	120	individuals with AUD and MetALD	placebo	Metabolic improvement from baseline as measured by percentage reduction in body weight and reduction in liver steatosis from baseline as measured by percentage reduction in Fibroscan controlled attenuation parameter (CAP) score.	July 2026
NCT06987513	Pemvidutide	sc, 2.4 mg once weekly	100	individuals with AUD and overweight or obesity	placebo	Change from baseline in the average number of heavy drinking days per week, with a heavy drinking day defined as 5 or more drinks in the day for men and 4 or more drinks in the day for women, using the TLFB method	June 2026
NCT07292519	Tirzepatide + Take Control CBT Module	sc, 5 mg once weekly	46	individuals with co-occurring AUD and overweight/obesity	placebo + Take Control CBT Module	Reduction in heavy drinking days, defined as 4 or more drinks in a day for women and 5 or more drinks in a day for men, using the TLFB method	January 2028
NCT07040592	Semaglutide	ND	30	individuals with AUD living with HIV	No control group	Proportion of participants who complete enrollment and duration of sessions. Number of sessions completed. Adherence to Medication. Safety of study assessed by adverse event reporting	June 2027
NCT07223983	Semaglutide	sc, 1 mg once weekly	10	individuals with AUD after metabolic and bariatric surgery with overweight or obesity	No intervention: wait-list control	Percent weight change. Mean drinks per calendar day to assess alcohol use. Mean drinks per drinking day to assess Alcohol use. Mean number of heavy drinking days to assess Alcohol use. Mean number of drinking vs. abstinent days to assess Alcohol use	October 2026
NCT06817356	Mazdutide	Administered sc, ND	300	individuals with AUD	placebo	Behaviors associated with AUD as assessed TLFB method	August 2026

Abbreviations: GLP-1, Glucagon-like peptide-1; AUD, Alcohol Use Disorder; sc, subcutaneously; po, per os; HIV, Human Immunodeficiency Virus; WHO, World Health Organization; MetALD, metabolic dysfunction and alcohol-associated liver disease; CAP, controlled attenuation parameter; TLFB, Timeline Follow Back; CBT, Cognitive–Behavioral Therapy; ND, no data; Source: ClinicalTrials.gov.

**Table 2:** Neural and molecular mechanisms mediating the effects of GLP-1 receptor agonists on addictive behaviors.

Drug Class	Substance	GLP-1RA	Key Brain Regions	Behavioral Effects	Mechanistic Insight	Ref.
Psychostimulants	Amphetamine	Ex-4	NAc	↓ AMPH-induced CPP. ↓ AMPH-induced locomotor activity	↓ AMPH-induced accumbal DA release	[112,113,114]
	Cocaine	Ex-4	VTA, NAc, LDTg, LS	↓ SA of cocaine ↓ cocaine-induced locomotor activity ↓ cocaine-induced CPP ↓ cocaine-induced reinforcement ↓ cocaine-seeking	↑ GABAergic inhibition ↓ cocaine-induced DA release ↑ DAT surface expression and function ↓ synthesis of pro-inflammatory cytokines by suppression of TLR4-dependent neuroimmune signaling	[112,113,114,115,116,117,118,119,120,121,124]
		Semaglutide	NAc	↓reinstatement of cocaine seeking ↓ SA of cocaine	↓ cocaine-induced DA levels in the NAc shell	[122]
Opioids	Oxycodone	Ex-4	NAc	↓ SA and reinstatement of oxycodone-seeking without diminishing its antinonciceptive effects	Modulation of D1R- and D2R-expressing MSNs	[129]
	Heroin	Liraglutide	NAc, VTA	↑ the latency to take heroin ↓ SA of heroin ↓ reinstatement of heroin-seeking	Putative central modulation of relapse-related motivational circuits	[130,131]
		Ex-4	NAc, VTA	↓ cue-induced heroin seeking ↓ reinstatement of heroin seeking (time-dependent) *	Modulation of orexin signaling in the NAc shell **	[134]
	Fentanyl	GEP44	ND	↓ fentanyl seeking without malaise during abstinence ↓ SA of fentanyl	Dual GLP-1R/Y2R activation	[133]
		Ex-4	ND	↓ reinstatement of fentanyl seeking ↓ SA of fentanyl	GLP-1R activation	[133]
Cannabinoids	Cannabis	Semaglutide	ND	↓ incident and recurrent CUD	CB1–GLP-1 interaction (observational)	[144]

Abbreviations: ↑, increased; ↓, decreased; ND, no data; AMPH, amphetamine; CB1, cannabinoid receptor type 1; CPP, conditioned place preference; CUD, cannabis use disorder; DA, dopamine; DAT, dopamine transporter; D1R, dopamine D1 receptor; D2R, dopamine D2 receptor; Ex-4, exendin-4; GABA, gamma-aminobutyric acid; GEP44, dual glucagon-like peptide-1 receptor/neuropeptide Y2 receptor agonist; GLP-1, glucagon-like peptide-1; GLP-1R, glucagon-like peptide-1 receptor; LDTg, laterodorsal tegmental nucleus; LS, lateral septum; MSNs, medium spiny neurons; NAc, nucleus accumbens; NF-κB, nuclear factor kappa B; OX1, orexin receptor type 1; SA, self-administration; TLR4, Toll-like receptor 4; VTA, ventral tegmental area; Y2R, neuropeptide Y2 receptor. * The time-dependent effect reflects suppression of drug-induced reinstatement after acute (1 h), but not delayed (6 h), Ex-4 administration. ** Authors suggested that Ex-4 reduced reinstatement of heroin-seeking via decreasing levels of orexin and compensatory increasing the Orexin 1 receptor (OX1) mRNA expression in the NAc shell.

## Data Availability

Not applicable.

## Abbreviations

The following abbreviations are used in this manuscript:

2-OG	2-oleoylglycerol
ACC	anterior cingulate cortex
AMPH	amphetamine
AUD	alcohol use disorders
AUDIT	Alcohol Use Disorder Identification Test
BMI	Body mass index
BNST	bed nucleus of the stria terminalis
CAP	controlled attenuation parameter
CB1	cannabinoid receptor type 1
CBT	Cognitive–Behavioral Therapy
CI	confidence interval
CPP	conditioned place preference
CUD	cannabis use disorder
CVD	cardiovascular disease
cAMP	cyclic adenosine monophosphate
DA	dopamine
DALYs	disability-adjusted life years
DAT	dopamine transporter
D1R	dopamine D1 receptor
D2R	dopamine D2 receptor
DMS	dorsomedial striatum
DPP-IV	dipeptidyl peptidase-4
EMA	European Medicines Agency
EU	European Union
EX-4	exendin-4
FDA	Food and Drug Administration
GABA	gamma-aminobutyric acid
GEP44	dual glucagon-like peptide-1 receptor/neuropeptide Y2 receptor agonist
GIP	glucose-dependent insulinotropic polypeptide
GLP-1	glucagon-like peptide-1
GLP-1R	glucagon-like peptide-1 receptor
GLP-1RA	glucagon-like peptide-1 receptor agonist
HIV	Human Immunodeficiency Virus
HR	hazard ratio
IL	interleukin
IPN	interpeduncular nucleus
LDTg	laterodorsal tegmental nucleus
LS	lateral septum
MHb	medial habenula
MetALD	metabolic dysfunction and alcohol-associated liver disease
MSNs	medium spiny neurons
NAc	nucleus accumbens
ND	no data
NF-κB	nuclear factor κB
NRT	nicotine replacement therapy
NTS	nucleus tractus solitarius
OEA	oleoylethanolamide
OFC	orbitofrontal cortex
OX1	orexin receptor type 1
PFC	prefrontal cortex
Po	per os
RCT	randomized clinical trial
SA	self-administration
Sc	subcutaneously
SNPs	single-nucleotide polymorphisms
T2D	type 2 diabetes
THC	Δ9-tetrahydrocannabinol
TLFB	Timeline Follow Back
TLR4	Toll-like receptor 4
TNF	tumor necrosis factor
VTA	ventral tegmental area
WHO	World Health Organization
Y2R	neuropeptide Y2 receptor
↑	increased
↓	decreased
